# Effectiveness of Oat-Hull-Based Ingredient as Fat Replacer to Produce Low Fat Burger with High Beta-Glucans Content

**DOI:** 10.3390/foods9081057

**Published:** 2020-08-04

**Authors:** Carmine Summo, Davide De Angelis, Graziana Difonzo, Francesco Caponio, Antonella Pasqualone

**Affiliations:** Department of Soil, Plant and Food Science (DISSPA), University of Bari Aldo Moro, Via Amendola, 165/a, I-70126 Bari, Italy; davide.deangelis@uniba.it (D.D.A.); graziana.difonzo@uniba.it (G.D.); francesco.caponio@uniba.it (F.C.); antonella.pasqualone@uniba.it (A.P.)

**Keywords:** beef burgers, soluble fiber, TPA, consumer evaluation, fatty acid composition

## Abstract

Low-fat beef burgers with high beta-glucan content was obtained using a gel made from an oat-hull-based ingredient as fat replacer. Two levels of fat substitution were considered: 50% (T1) and 100% (T2). The nutritional composition, cooking yield, textural properties, color characteristics and consumer preference were evaluated, in comparison with a burger without fat replacer (CTRL). After cooking, T2 burger showed a significant increase in the cooking yield and a very low lipid content (3.48 g 100 g^−1^) as well as a level of beta-glucans per single portion (2.96 g 100 g^−1^) near the recommended daily intake. In T1 burger, the decrease of lipid content was mitigated during the cooking process, because the beta-glucans added had a fat-retaining effect. Compared to CTRL, replacing fat led to a softer texture of cooked burgers evaluated by Texture Profile Analysis. The differences in color, significant in raw burgers, were smoothed with cooking. The consumer evaluation, carried out according to the duo-trio test, highlighted significant differences between CTRL and T2 burgers in terms of odor, taste, color and texture. The consumers expressed a higher preference for the T2 burger, probably due to its softer texture and greater juiciness.

## 1. Introduction

Meat and meat products play an important role in human nutrition, constituting a rich source of proteins with high biological value, vitamins (A, B1, B3 and B12), as well as iron, zinc and other micronutrients [1]. The consumption of meat and meat products dates back to antiquity, but these products are still part of the gastronomic tradition of many countries. Therefore, a high number of Protected Designation of Origin (PDO) and Protected Geographical Indication (PGI) European brands—which link the quality of food products to a specific geographical area—have been awarded to “Meat products—cooked, salted and smoked” (205 registered products, accounting for 12.95% of the total PDO and PGI products) and “Fresh meat and offal” (180 registered products, i.e., 11.37% of the total PDO and PGI products) [2]. However, the high fat content of meat products (including saturated fatty acids and cholesterol) is related to increased risk of developing coronary heart diseases [3].

In this context, researchers and private companies alike are strongly engaged in trying to improve the nutritional value of meat products by lowering the cholesterol and lipid content, as well as decreasing saturated and increasing polyunsaturated fatty acids. Fats, however, play an important role in meat products, ensuring optimal rheological and textural properties [4] and conferring pleasant sensorial characteristics in terms of flavor and juiciness [5]. Therefore, the reduction of lipid content in meat involves the use of ingredients able to mimic the properties of fat, such as polysaccharides. Several experimental trials have therefore been performed that included various mostly fiber-rich polysaccharide-based fat replacers in the formulation of meat products, such as ground poppy seeds [6], mixtures of wheat fiber and pig skin [7], legume flours [8] and other vegetable sources, as indicated in recent reviews [9]. Dietary fiber can form a compact gel due to the ability to bind water improving the structural characteristics of reduced-fat products [10].

Among dietary fibers, beta-glucans from cereal grains have been recently studied in relation to the health benefits associated with their consumption such as the reduction of cholesterol level and a chemo-preventive effect as reported by Ho et al. [11].

Moreover, beta-glucans show several technologically useful properties (gelling capacity, emulsifying activity, fat/water binding capacity), which make them suitable ingredients in health-promoting functional foods [12]. The major applications of beta-glucans in food formulation are in milk-based products, such as fermented milk products and yogurt [13] and in bakery products [14]. Several beta-glucan sources have also been considered for improving the nutritional quality of meat products, with [15,16,17] or without [18] fat replacement. However, the level of beta-glucan enrichment reported in previous studies on meat products does not reach the recommended daily intake for beta-glucans, which accounts for 3 g per day [19].

In this frame, the aim of this study was the production of low-fat burgers with a beta-glucan content very close to the recommended daily intake and with good textural and sensorial characteristics.

## 2. Materials and Methods

### 2.1. Preparation of the Fat Replacer

An oat-hull-based ingredient (Nutraceutica S.R.L., Monterenzio, Italy) containing, as declared by the producer, 55% beta-glucans, <10% proteins, <2% fat, was used to prepare a gel by mixing 27.27 g of flour with 72.73 mL of distilled water for 5 min at 13,500 rpm by means of a T25 Ultraturrax (IKA, Staufen, Germany). The gel was then cut into small pieces to be used, freshly prepared, as a fat replacer in burgers.

The ratio flour:water was defined in preliminary tests to obtain a gel: (i) able to mimic as much as possible the consistency and homogeneity of the beef fat conventionally used to prepare meat burgers; (ii) having a beta-glucan concentration able to achieve, when added to burgers as total fat replacer, a beta-glucan content as near as possible to the daily intake recommendation (3 g per day) [19].

### 2.2. Preparation of the Beef Burgers

Beef meat, purchased at a local butcher’s shop, was manually sectioned with a sharp knife to separate the lean meat from the visible adipose and connective tissues. Then, the lean meat (3.5 g 100 g^−1^ fat content) and the adipose tissue (71.5 g 100 g^−1^ fat content, still containing residual proteins and moisture) were separately ground using a grinder equipped with a 4 mm plate (Kenwood MG510, Delonghi Appliances, Treviso, Italy). Adipose tissue and lean meat, both ground, were then mixed manually. During the mixing step, three batches were prepared, according to three different formulations at increasing levels of fat: control (CTRL), with 15% of beef adipose tissue added; T1, with a partial (50%) substitution of beef adipose tissue (i.e., with 7.5% beef adipose tissue and 7.5% oat-hull-based gel added); and T2, with a total substitution of beef adipose tissue (i.e., with 15% oat-hull-based gel added). With the exception of salt, no other spices or ingredients were added. The formulations of the three burgers are reported in Table 1. The burgers, weighing approximately 50 g, were finally shaped (70 mm diameter, 10 mm thickness) using a burger maker mold. The whole experiment was repeated twice.

### 2.3. Cooking Procedure

The burgers were cooked according to the American Meat Science Association methodology [20], i.e., were roasted in an electric oven (Delonghi EO 3275, Delonghi Appliances, Treviso, Italy) preheated at 163 °C, until their internal temperature, measured by a digital thermometer (LT−101, TFA Dostmann, Reicholzheim, Germany), reached 71 °C. Approximatively 10 min was sufficient to cook all the samples perfectly.

Cooked burgers were then submitted to the chemical and textural determinations, as well as consumer test. The colorimetric determinations, instead, were carried out on burgers both before (raw) and after cooking.

### 2.4. Chemical Composition of Beef Burgers

Protein content (total nitrogen × 6.25), ash, and moisture content were determined, according to the AOAC International methods, to be 928.08, 920.153 and 950.46, respectively [21]. The lipid content was determined by Folch method [22] using chloroform and methanol (Sigma Aldrich, Milan, Italy) as extracting solvent. The carbohydrate content was determined as difference. The total beta-glucan concentration was determined by the AOAC International method 995.16 [23] by using the Megazyme mixed-linkage beta-glucan assay kit (Megazyme International, Bray, Ireland). The total energy value for each product was calculated by using the Atwater coefficients as reported in Summo et al. [24]. All determinations were carried out in triplicate.

### 2.5. Fatty Acid Composition of Beef Burgers

The fatty acid composition was determined by gas-chromatographic (GC) analysis of fatty acid methyl esters. The lipid fraction was cold-extracted with methanol/chloroform (1:2 *v*/*v*) following the method proposed by Folch et al. [22]. The methylation was carried out according to the AOCS (American Oil Chemists Society) method Ch 1–91 [25]. The GC system and conditions were the same as those reported in a previous paper [26]. The identification of each fatty acid was carried out by comparing the retention time with that of the corresponding methyl ester standard (Sigma Aldrich, Milan, Italy). All determinations were carried out in triplicate.

Atherogenic (AI) and Thrombogenic (TI) indices were calculated according to the following equations [27]:

AI = [C_12:0_ + (4 × C_14:0_) + C_16:0_]/(n-6 PUFA + n-3 PUFA + MUFA) (1)

TI = (C_14:0_ + C_16:0_ + C_18:0_)/[0.5 × MUFA + 0.5 × n-6 PUFA + 3 × n-3 PUFA + (n-3 PUFA/n-6 PUFA)] (2)

where PUFA are polyunsaturated and MUFA monounsaturated fatty acids. C_12:0_, C_14:0_, C_16:0_ andC_18:0_ are lauric, myristic, palmitic and stearic acids, respectively.

### 2.6. Cooking Yield

The cooking yield of beef burgers was determined by measuring the weight (w) of the burgers before and after cooking according to the following equation:

Cooking yield = (w cooked burger/w raw burger) × 100.(3)

The calculation has been performed on ten burgers.

### 2.7. Texture Profile Analysis

Texture profile analysis (TPA) of beef burgers was performed according to Afshari et al. [17] with some modifications, using a texture analyzer model Z1.0 TN (Zwick Roell, Ulm, Germany) equipped with a 3.6 cm cylindrical probe and a 1 kN load cell. The samples were heated in an oven at 60 °C in order to simulate the serving conditions. Then, a portion of 2 cm of diameter was cut from the center of the burger. A two-compression cycle was carried out at the speed of 5 mm s^−1^, with 5 s of pause between the two compressions, up to 70% of recorded deformation. The following parameters were assessed: hardness (N), indicating the maximum force recorded during the first compression; cohesiveness, measured as the area of work during the second compression divided by the area of work during the first compression; gumminess (N), calculated as hardness × cohesiveness; springiness, measured by the distance of the detected height during the second compression divided by the original compression distance; chewiness (N), calculated as gumminess × springiness. Ten different burgers per formulation were considered, and each burger was subjected to one measurement by TPA.

### 2.8. Color Determination of Burgers

Instrumental determination of the surface color of both raw and cooked burgers was carried out by using the CM-600d colorimeter (Konica Minolta, Tokyo, Japan) supported by SpectraMagic NX software (Konica Minolta, Tokyo, Japan). The CIE (International Commition on Illumination) L*, a*, and b* parameters were recorded: lightness (L*), red index (a*) and yellow index (b*), together with ΔE [28].

∆E = [(∆L*)2 + (∆a*)2 + (∆b*)2]^1/2^ (4)

Three samples per formulation were analyzed, and four readings were recorded in different areas of each sample.

### 2.9. Duo-Trio Consumer Test

CTRL and T2 burgers were submitted to consumer test according to the duo-trio test methodology [29] to determine if the differences between them could be recognized. Sixty people, regular consumers of meat and neither food-allergic nor intolerant, were recruited among the researchers and students of the Agricultural Faculty of the University of Bari Aldo Moro (Bari, Italy). The study protocol followed the ethical guidelines of the laboratory. Each participant was given information about study aims and individual written informed consent was obtained from each participant. The consumer test was performed at a local restaurant sited in Bari (Italy). Each participant received three samples on the same dish: one as reference (CTRL or T2 randomly, and codified with an alphanumeric code), and the other two were both CTRL and T2 randomly distributed, codified with an alphanumeric code. Each consumer was asked to indicate the sample that was different respect to the reference in terms of color, odor, taste and texture. Moreover, each panelist expressed a judgment indicating which burger preferred. The results were expressed as number of correct answers.

### 2.10. Statistical Analysis

Data were subjected to one-way ANOVA followed by the Tukey’s HSD test. Significant differences were determined at *p* < 0.05 by the XLStat software (Addinsoft SARL, New York, NY, USA).

The results of duo–trio test were expressed as number of correct answers considering thirty-nine, forty-one and forty-four as minimum correct answers to identify statistically significant differences at *p* < 0.05, *p* < 0.01 and *p* < 0.001, respectively [30].

## 3. Results and Discussion

### 3.1. Chemical Composition

The addition of the fat replacer significantly influenced the chemical composition of cooked burgers (Table 2). An increase of moisture was observed at increasing content of fat replacer. This is principally due to the high moisture content of the fat replacer. These findings agreed with those of a previous study involving the use of oat beta-glucan as fat replacer [16]. However, in another study, the use of gelled emulsion (based on olive oil, gelatin and 9% inulin) caused an increase of moisture content only in raw patties, whereas a significantly lower moisture of cooked product was observed due to lower cooking yield and water holding capacity of the gel [31]. Therefore, our results could be due also to better moisture retention of fat-substituted burgers during cooking due to the high hydrophilicity of beta-glucans [32], able to increase the water-holding capacity of the product. The total substitution of fat (T2), indeed, caused a significantly higher moisture content than in CTRL and T1.

On the contrary, the protein content of beef burgers (on fresh matter), showed a progressive and significant decrease when the fat replacement increased. Piñero et al. [15] and Afshari et al. [17] reported that the addition of a beta-glucan-based fat replacer had no significant influence on the protein content. Our findings could be related to a higher level of gel incorporation and a consequently higher moisture content. Moreover, the beef adipose tissue used in CTRL and T2 formulations contained muscular residues, which also contributed to the protein content, in accordance with other authors [33]

Compared to CTRL, the addition of the fat replacer resulted in a slight but significant fat decrease in T1 formulation, whereas the T2 burger showed a more marked decrease. Considering the lipid content of the beef adipose tissue (accounting for 71.5%) used in CTRL and T1 formulations, and the contribution of the residual intramuscular fat of the lean fraction (3.5%), the lipid content of the CTRL raw burger could be estimated at 13.6 g 100 g^−1^. After cooking, the CTRL burger showed a lipid content of 8.42% (6.04 g of fat in 71.82 g of cooked burgers); therefore, an estimated fat loss of 56% occurred. The lipid content of the raw T1 burger could be estimated at 8.17 g 100 g^−1^, whereas the cooked burger had a 7.25% fat content (5.45 g of fat in 75.14 g of cooked burger), with a fat loss of 34%. Therefore, even if considering estimated values, cooking induced a more limited fat loss when fat was replaced by the beta-glucan based gel than in the CTRL burger. This phenomenon could be imputable to the ability of the beta-glucans to form a tri-dimensional network which entraps fat and water within the meat protein system [15]. Therefore, it has to be considered that partial fat replacement with beta-glucans lowers fat content in the raw product, but this nutritionally positive effect is mitigated by higher fat retention during the cooking process. As a consequence, a total fat replacement has to be made to achieve a significant nutritional effect on the cooked product.

The use of the fat replacer caused, as expected, a slight but significant increase in the carbohydrate content of T1, even if no significant differences were observed comparing T1 and T2. This was imputable to the presence of carbohydrates in the oat-hull-based ingredient. The addition of vegetable fat replacer in burgers is reported to be influential on the chemical composition of the product [34]. The content of beta-glucans reached a level that made the health claim “beta-glucans contribute to the maintenance of normal blood cholesterol levels” applicable to both T1 and T2 burgers since the concentration of these compounds was always higher than 1 g per recommended portion (in meat products, this quantity corresponds to 100 g). However, the claim regulation specifies that “the beneficial effect is obtained with a daily intake of 3 g of beta-glucans” [19]. In this regard, T2 burger contained 2.96% of beta-glucans. Therefore, the recommended daily intake of beta-glucans, according to the above-mentioned regulation, could be reached by consuming a single portion (100 g) of T2 burger. This result is particularly important because it is possible to achieve a significant improvement in the nutritional characteristics of burgers. Indeed, by combining the total substitution of animal fat with the inclusion of functional macromolecules, a positive effect on cholesterol reduction could be expected. Indeed, it is known that beta-glucan has an active role on the reduction of LDL-cholesterol [11] by modulating the cholesterol metabolism and the gut microbiota [35].

The fat substitution resulted in a significant decrease in energy value, from 203.44 kcal 100 g^−1^ (CTRL) to 146.24 kcal 100 g^−1^ (T2). In particular, the T2 formulation allowed the research to obtain a product with lower fat content and, consequently, lower energy value compared to the products proposed by other studies [17,18,19]. An effective improvement of the nutritional value of meat products was therefore achieved, due to reduced fat content, relatively low energy value and high concentration of beta-glucans.

### 3.2. Cooking Yield

The fat replacement caused an increase in cooking yield. The difference, compared with the control burger, became significant in the T2 formulation. These findings agreed with previous studies [17,36] in which higher cooking yield and moisture retention with the increase of beta-glucan content was observed. This behavior can be explained with the already mentioned ability of beta-glucans to form three-dimensional structures with meat proteins, which can easily entrap water and fat, increasing the cooking yield [15].

### 3.3. Fatty Acid Composition

Fatty acid composition of burgers is reported in Table 3, as mg 100 g^−1^ of burger and g 100 g^−1^ of fatty acids. The nutritional value of beef burgers is also related to the composition of the lipid fraction, which usually is dominated by saturated fatty acids, palmitic and stearic acids in particular, whereas oleic acid was the most abundant unsaturated acid. The fatty acid composition of cooked burgers agreed with other studies carried out on the same category of products [17,37]. Owing to the fat substitution, a significant reduction was observed of the quantity (mg 100 g^−1^ of burger) of all fatty acids due to the general decrease of lipid content. Moreover, a different level of reduction was observed as a function of the unsaturation rate. In particular, T2 showed a content of palmitic acid 60% lower than the CTRL. The reduction was slightly lower for oleic acid (−57%), whereas linolenic, the most abundant polyunsaturated fatty acid, decreased by 45% comparing T2 with CTRL. This aspect could be better explained considering the composition of fatty acids expressed as percentage. In particular, comparing the T2 with the other formulations, we observed a significantly (*p* < 0.05) lower percentage of saturated fatty acids and a higher percentage of the polyunsaturated fatty acids, whereas the monounsaturated fatty acids remained constant across the formulations. Previous studies report significant differences in the fatty acid composition of subcutaneous and muscular beef fat, with the latter characterized by higher polyunsaturated and lower saturated fatty acids [38,39]. This could explain the differences observed in our samples, because in CTRL and T1 burgers, the fatty fraction added was mainly subcutaneous fat, while in T2 the residual fat was constituted principally by muscular fat.

Albeit in low amounts, we detected also some polyunsaturated fatty acids important from a nutritional point of view, such as the arachidonic (C_20:4 n-6_) eicosapentaenoic (C_20:5 n-3_) and docosapentaenoic acids (C_22:5 n-3_), without significant differences among the formulations. The amount of these important fatty acids was lower than that reported in other studies carried out on the raw beef lipid fraction [40]. This difference could be related to the cooking procedure, which causes the loss of these fatty acids [37]. In studies carried out on cooked beef burgers, these fatty acids were indeed not determined [17,41].

As a consequence of the different lipid composition, the nutritional indices linked to the fatty acid composition were also influenced by the fat replacement. In particular, the PUFA/SFA ratio significantly increased in T2 compared to CTRL. Moreover, the atherogenic and thrombogenic indices related to fatty acid composition significantly decreased in T2 burger with 100% fat substitution, although the values were higher than those recommended [42]. The n-6/n-3 ratio was higher in T2 compared to CTRL and T1. It is reported that lowering the n-6/n-3 ratio to less than 4 is desirable to improve the healthiness of the product [43,44]. However, the achievement of this target in meat product is not possible solely with a fat reduction, because fat composition needs to be reformulated by the addition of oils rich in n-3 PUFA [44,45].

Similar improvements were observed by Pintado et al. [45] in fresh sausages obtained using an olive oil in water emulsion containing chia and oat as fat replacer. The authors explained the results with the high level of polyunsaturated fatty acids of chia. The oat-hull-based ingredient used in our study was characterized by a very low lipid content; therefore, its contribution to the fatty acid composition was of relevance. Several studies report that the unsaturated fatty fractions are combined with structural compounds of meat so that their loss during cooking is less influenced than saturated fatty acids [44]. The saturated fatty acids could easily be lost during cooking, and this could explain the observed results.

### 3.4. Texture Profile Analysis

Significant differences in the textural properties were observed among burgers with different formulation (Table 4). The incorporation of a fat replacer led to a significant decrease of hardness, cohesiveness, gumminess and chewiness in T1 and T2 burgers compared to CTRL, indicating that these burgers had a softer texture and then required less energy to be compressed. No significant differences, however, were found between T1 and T2, highlighting the fact that the level of fat substitution did not influence the textural properties of beef burgers.

The trend of moisture and fat as an influence on texture [17] could be explained by a compensation between the differences in moisture and fat contents of T1 and T2 (Table 2), leading to similar textural properties. The effect of the fat substitution level was significant only for springiness, which showed the lowest value in T2 formulation.

Owing to the important structural functions of fat, the influence on the textural properties should be considered when the target of a new food formulation is fat substitution. The use of beta-glucans as fat replacement in beef burger or beef patties was previously studied by other authors with contrasting results, depending on whether beta-glucans were added as powder, gel or emulsion. In particular, Szpicer et al. [16] reported an increase in hardness of meat burgers after the addition of 30% beta-glucan concentrate powder. When the beta-glucans were added as gel [15] or emulsion [36], a significant reduction of hardness and other textural parameters were observed. With the increase of beta-glucans concentration, the amount of water available for proteins decreases and meat products lose springiness [46]. This behavior could be explained by a higher moisture retention of burgers and a consequently lower compactness of protein matrix [36]. Furthermore, beta-glucans have the ability to bind not only water but also fat, allowing the formation of a softer [47] and juicier product [17].

### 3.5. Color Indices

Color evaluations on the raw burger were made because the color characteristics of the meat products can influence the consumers’ willingness to purchase, with increasing appreciation for bright red products. In raw burgers, a progressive and significant increase of lightness (L*) and yellowness (b*) was observed with fat replacement, while redness (a*) was not significantly influenced (Table 5). The increase of the lightness and yellowness could be related to the presence of yellow pigments such as lutein in oat (the source of beta-glucan enriched gel), as previously reported in [48]. In contrast, a* remained constant, indicating that the fat substitution was not significant on this index. Moreover, in a previous study, the fat substitution with a chia oil emulsion gel caused no significant variations of a* but significant changes of L* and b* [49]. In the same study, L* and b* were slightly higher than ours, probably because of the presence of the oil in the fat replacer.

The differences observed among raw burgers were smoothed by cooking, after which no significant differences were found for all the color indices, as reported also by Gök et al. [6]. The color of burgers reformulated with fat replacers is influenced by the type of ingredients used for this purpose. In particular, Lucas-González et al. [49] reported a decrease of L* and an increase of a* during cooking of burgers formulated with chestnut flour and chia oil emulsion gels. By contrast, Heck et al. [43] reported an increase of L* and a decrease of a* in cooked burgers produced by the inclusion of linseed or chia oil microparticles. During the cooking process, meat color changes due to the heat-induced denaturation of myoglobin. Our results, assessed on the cooked burgers, were not influenced by fat substitution; however, it is reasonable to say that the primary contribution to color is given by meat. The role of fat in influencing the color of cooked meat is not fully understood [50], but it should have a lower influence on color than other critical parameters, such as pH and storage conditions [50].

The ΔE of T1 and T2 formulations, calculated by comparing them to the CTRL, was determined in order to improve evaluation of the color differences between samples. The ΔE was higher in raw than in cooked burgers, reaching the maximum of 7.16 in T2 formulation, whereas T1 showed a value of 3.89. ΔE values were between 3.5 and 5.0, meaning that the observer can clearly perceive the difference between samples; thus, T1 raw burgers could be easily distinguished from CTRL. ΔE values higher than 5 indicate the presence of two distinct colors [51]. When considering the cooked burgers, a decrease of ΔE of both T1 and T2 was observed. The changes occurring in T2 burger were particularly interesting due to the drop of ΔE at 2.58. When 2.0 < ∆E < 3.5, even an unexperienced observer can notice the difference in color between products [51].

### 3.6. Consumer Test

CTRL and T2 were submitted to a consumer test, according to the duo–trio test methodology [28], which was chosen to determine if the differences between burgers in terms of color, odor, taste and texture were recognizable by consumers. T1 burger was not considered for two main reasons. Firstly, after preliminary sensory analysis, a small group of trained panelists agreed that T1 burger was similar to CTRL. Moreover, considering the nutritional characteristics of T2 burgers, they were noticeably more interesting than T1, therefore we selected only T2 burger, which had no fat added and had a high content of beta-glucans.

**Figure 1:**
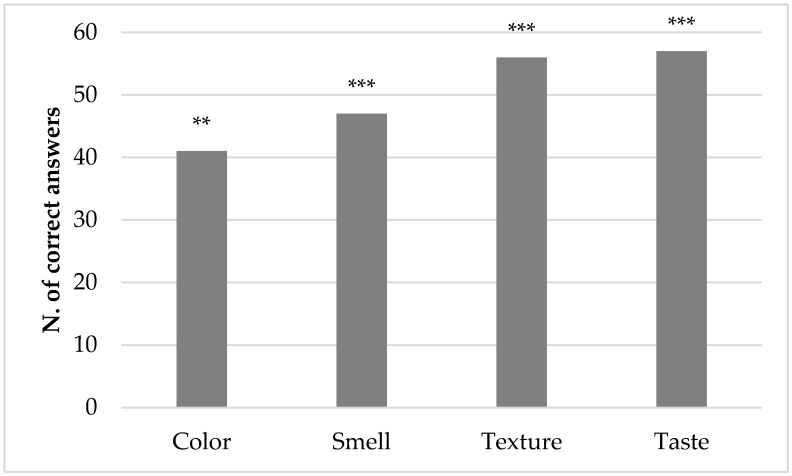
Number of people recognizing the difference between burger without fat substitution (CTRL) and at 100% fat substitution (T2) in a duo–trio consumer test. **: significance *p* < 0.01; ***: significance *p* < 0.001.

As shown in Figure 1, the consumers recognized the difference between CTRL and T2 burgers for all the descriptors. In particular, forty-one people recognized CTRL and T2 for their different color (*p* < 0.01), whereas the number of correct answers increased when considering odor, texture and taste, with highly significant results (*p* < 0.001). The consumer test confirmed the results of textural and colorimetric evaluations (see for example the ΔE parameter). Szpicer et al. [16] also reported that consumers could distinguish products containing fat replacers, based on differences in color, texture, aroma and taste. Moreover, Afshari et al. [17] highlighted that fat substitution was perceived as significantly different by sensory analysis. On the whole, the substitution of fat with the beta-glucan gel changed the textural and sensorial quality of burgers, but the modification did not cause a deterioration of the general appreciation of products. In actual fact, 59.32% of panelists expressed a preference for T2 burger, and 40.68% preferred the CTRL burger. This difference was devoid of statistical significance (*p* > 0.05); therefore, the addition of beta-glucan gel did not cause a significant decrease in the sensorial acceptability of the burgers. Both texture and taste, in fact, are known to influence the acceptability of meat products, especially the juiciness and the tenderness [52]. Moreover, as reported by Desmond et al. [53], a low water binding capacity implicates a negative effect on palatability, due to the lack of juiciness and brittle texture which are both generally unacceptable to the consumers.

## 4. Conclusions

The use of an oat-hull-based gel as fat replacer allowed us to obtain a beef burger with a very low lipid content (3.48 g 100 g^−1^ in the formulation with a total fat substitution) and with a 2.96 g 100 g^−1^ content of beta-glucans, almost reaching the recommended daily intake per single portion of burger. With a partial substitution, the decrease of lipid content in the raw product was mitigated during the cooking process (34% and 56% of estimated fat loss in T1 and CTRL respectively). This could be related to the fat-retaining effect of beta-glucans added. Compared to CTRL, replacing fat by the oat-hull-based gel caused a significant decrease in hardness and other textural parameters of cooked burgers. Conversely, the differences in color, significant in raw burgers, were smoothed with cooking. The consumer evaluation, carried out according to the duo–trio test, highlighted significant differences between CTRL and T2 burgers in terms of odor, taste, color and texture. The consumers expressed a higher preference for the T2 burger, probably due to its softer texture and greater juiciness.

These results are a step forward for the improvement of the nutritional characteristics of meat products and indicate that the use of the oat-hull-based ingredient, rich in beta-glucans, as gel is an effective strategy for a complete fat substitution.

## Figures and Tables

**Table 1:** Formulation (g kg^−1^) of three different beef burgers without fat substitution (CTRL) and at 50% (T1) and 100% (T2) fat substitution.

Ingredient/Formulation	Samples		
	CTRL	T1	T2
Beef lean meat	835.25	835.25	835.25
Beef adipose tissue	150.00	75.00	0
Oat hull based gel *	0	75.00	150.00
Salt	14.75	14.75	14.75

* Gel as fat replacer formulated with 27.27 g of oat hull ingredient at 55% of beta-glucan concentration emulsified with 72.73 mL of distilled water.

**Table 2:** Chemical composition, cooking yield and energy value of the cooked beef burger without fat substitution (CTRL) and at 50% (T1) and 100% (T2) fat substitution with an oat-hull-based gel.

	CTRL	T1	T2	*p*-Value
Moisture (% f.w.)	57.24 ± 0.22^C^	58.79 ± 0.46^B^	63.39 ± 0.26^A^	*p* < 0.001
Protein (% f.w.)	28.41 ± 0.29^A^	26.98 ± 0.15^B^	25.83 ± 0.10^C^	*p* < 0.001
Fat (% f.w.)	8.42 ± 0.04^A^	7.25 ± 0.12^B^	3.48 ± 0.03^C^	*p* < 0.001
Ash (% f.w.)	2.42 ± 0.31^AB^	2.29 ± 0.25^B^	2.93 ± 0.16^A^	*p* = 0.045
Total Carbohydrates (% f.w.)	3.51 ± 0.43^B^	4.70 ± 0.43^A^	4.38 ± 0.49^AB^	*p* = 0.044
Beta-glucan (% f.w.)	0.01 ± 0.01^C^	1.35 ± 0.13^B^	2.96 ± 0.07^A^	*p* < 0.001
Cooking Yield (%)	71.82 ± 1.39^B^	75.14 ± 2.13^B^	80.30 ± 2.50^A^	*p* = 0.007
Energy Value (kcal/100 g)	203.44 ± 0.13^A^	186.47 ± 3.49^B^	146.24 ± 1.88^C^	*p* < 0.001

Data on the chemical composition were expressed as % on fresh (f.w.) weight. Different letters in the same row indicate significant differences at *p* < 0.05.

**Table 3:** Fatty acid composition (g 100 g^−1^ of burger and g 100 g^−1^ of fatty acids) and the nutritional index of the beef burger without fat substitution (CTRL) and at 50% (T1) and 100% (T2) of fat substitution with an oat-hull-based gel.

	mg 100 g^−1^ of Burger			g 100 g^−1^ of Total Fatty Acids		
	CTRL	T1	T2	CTRL	T1	T2
Myristic C_14:0_	395.29 ± 12.98^A^	300.95 ± 20.07^B^	111.52 ± 4.78^C^	*4.69± 0.15^A^*	*4.15 ± 0.28^B^*	*3.20 ± 0.14^C^*
Myristoleic C_14:1_	115.74 ± 7.41^A^	75.77 ± 0.59^B^	37.93 ± 5.14^C^	*1.37 ± 0.09^A^*	*1.05 ± 0.01^B^*	*1.09 ± 0.15^B^*
Pentadecanoic C_15:0_	45.57 ± 0.37^A^	38.12 ± 0.94^B^	17.44 ± 0.85^C^	*0.54 ± 0.00^A^*	*0.53 ± 0.01^A^*	*0.50 ± 0.02^A^*
Pentadecenoic C_15:1_	14.58 ± 0.82^A^	12.06 ± 2.50^A^	8.23 ± 0.03^B^	*0.17 ± 0.01^B^*	*0.17 ± 0.03^B^*	*0.24 ± 0.00^A^*
Palmitic C_16:0_	2300.53 ± 31.67^A^	1985.39 ± 53.88^B^	934.19 ± 14.64^C^	*27.32 ± 0.38^A^*	*27.38 ± 0.74^A^*	*26.84 ± 0.42^A^*
Palmitoleic C_16:1_	460.47 ± 15.01^A^	370.8 ± 14.13^B^	173.9 ± 1.34^C^	*5.47 ± 0.18^A^*	*5.11 ± 0.19^AB^*	*5.00 ± 0.04^B^*
Heptadecanoic C_17:0_	72.67 ± 1.48^A^	71.02 ± 2.33^A^	28.22 ± 0.99^B^	*0.86 ± 0.02^B^*	*0.98 ± 0.03^A^*	*0.81 ± 0.03^B^*
Heptadecenoic C_17:1_	56.71 ± 0.17^A^	57.06 ± 2.17^A^	32.72 ± 0.92^B^	*0.67 ± 0.00^C^*	*0.79 ± 0.03^B^*	*0.94 ± 0.03^A^*
Stearic C_18:0_	1146.61 ± 29.63^A^	1067.91 ± 18.60^B^	485.95 ± 5.80^C^	*13.62 ± 0.35^B^*	*14.73 ± 0.26^A^*	*13.96 ± 0.17^B^*
Oleic C_18:1 n-9_	3252.74 ± 56.21^A^	2939.81 ± 113.70^B^	1361.05 ± 45.98^C^	38.63 ± 0.67^A^	40.45 ± 1.57^A^	39.11 ± 1.32^A^
Linoleic C_18:2 n-6_	409.06 ± 19.51^A^	266.87 ± 34.27^B^	236.6 ± 22.44^B^	4.86 ± 0.23^B^	3.68 ± 0.47^B^	6.80 ± 0.64^A^
Linolenic C_18:3 n-6_	39.67 ± 4.77^A^	30.33 ± 1.23^B^	11.26 ± 0.42^C^	*0.47 ± 0.06^A^*	*0.42 ± 0.02^A^*	*0.32 ± 0.01^B^*
dihomo-γ-linolenic C_20:3 n-6_	58.61 ± 7.50^A^	17.20 ± 5.95^B^	23.55 ± 2.43^B^	*0.70 ± 0.09^A^*	*0.24 ± 0.08^B^*	*0.68 ± 0.07^A^*
Arachidonic C_20:4 n-6_	29.96 ± 11.23^A^	9.61 ± 3.47^B^	9.41 ± 0.95^B^	*0.36 ± 0.13^A^*	*0.13 ± 0.05^B^*	*0.27 ± 0.03^AB^*
Eicosapentaenoic C_20:5 n-3_	9.77 ± 2.08^A^	3.29 ± 1.75^B^	3.40 ± 2.95^B^	*0.12 ± 0.02^A^*	*0.08 ± 0.02^A^*	*0.10 ± 0.03^A^*
Docosapentaenoic C_22:5 n-3_	12.03 ± 0.88^A^	3.82 ± 0.49^C^	5.48 ± 0.41^B^	*0.14 ± 0.03^A^*	*0.12 ± 0.02^A^*	*0.16 ± 0.03^A^*
ƩSFA	3960.67 ± 16.87^A^	3463.39 ± 53.96^B^	1577.32 ± 13.74^C^	47.04 ± 0.20^A^	47.77 ± 0.74^A^	45.33 ± 0.39^B^
ƩMUFA	559.09 ± 45.97^A^	331.13 ± 44.70^B^	289.70 ± 27.59^B^	46.32 ± 0.75^A^	47.56 ± 1.36^A^	46.37 ± 1.19^A^
ƩPUFA	3900.24 ± 62.84^A^	3455.48 ± 98.65^B^	1613.83 ± 41.29^C^	6.64 ± 0.55^B^	4.67 ± 0.62^C^	8.32 ± 0.79^A^
MUFA/SFA ratio				0.98 ± 0.02^A^	1.00 ± 0.04^A^	1.02 ± 0.04^A^
PUFA/SFA ratio				0.14 ± 0.01^B^	0.10 ± 0.01^C^	0.18 ± 0.02^A^
AI				0.87 ± 0.02^A^	0.88 ± 0.05^A^	0.73 ± 0.02^B^
TI				1.68 ± 0.01^A^	1.71 ± 0.05^A^	1.57 ± 0.02^B^
n-6/n-3 PUFA				24.78 ± 1.41^A^	22.35 ± 9.47^A^	33.35 ± 8.89^AB^

SFA = Saturated fatty acids; MUFA = Monounsaturated fatty acids; PUFA = Polyunsaturated fatty acids; AI = Atherogenic Index; TI = Thrombogenic Index. Different letters in the same row indicate significant differences at *p* < 0.05.

**Table 4:** Texture profile analysis (TPA) of the beef burger without fat substitution (CTRL) and at 50% (T1) and 100% (T2) of fat substitution with an oat-hull-based gel.

	Hardness (N)	Springiness	Gumminess	Chewiness (N)	Cohesivity (N)
CTRL	159.1 ± 10.4^A^	0.71 ± 0.02^A^	56.2 ± 7.7^A^	40.2 ± 6.4^A^	0.35 ± 0.04^A^
T1	116.0 ± 7.5^B^	0.68 ± 0.02^B^	33.9 ± 2.7^B^	23.0 ± 2.3^B^	0.29 ± 0.01^B^
T2	113.7 ± 9.8^B^	0.62 ± 0.03^C^	29.8 ± 3.8^B^	18.5 ± 2.7^B^	0.26 ± 0.02^B^
*p*-Value	*p* < 0.001	*p* < 0.001	*p* < 0.001	*p* < 0.001	*p* < 0.001

Different letters in the same column indicate significant differences at *p* < 0.05.

**Table 5:** Instrumental color determination of the beef burger without fat substitution (CTRL) and at 50% (T1) and 100% (T2) of fat substitution with an oat-hull-based gel before (Raw) and after (Cooked) cooking.

		Raw			Cooked	
	CTRL	T1	T2	CTRL	T1	T2
L*	39.04 ± 0.77^C^	41.07 ± 0.30^B^	42.97 ± 1.28^A^	48.00 ± 2.21^A^	48.22 ± 2.00^A^	47.69 ± 1.41^A^
a*	13.63 ± 0.40^A^	13.40 ± 0.63^A^	14.15 ± 1.76^A^	6.09 ± 0.91^A^	6.22 ± 0.73^A^	6.23 ± 0.41^A^
b*	14.73 ± 0.32^C^	17.84 ± 0.18^B^	20.35 ± 2.17^A^	13.26 ± 1.30^A^	11.60 ± 0.86^AB^	12.18 ± 0.67^B^
∆E _vs. CTRL_		3.89 ± 0.36	7.16 ± 2.61		3.38 ± 1.55	2.58 ± 1.62

Different letters in the same row indicate significant differences at *p* < 0.05.
